# Polyethylene glycol 3350 plus electrolytes for chronic constipation: a 2-week, randomized, double-blind, placebo-controlled study with a 52-week open-label extension

**DOI:** 10.1007/s00535-019-01581-x

**Published:** 2019-04-22

**Authors:** Atsushi Nakajima, Kazuhiko Shinbo, Akira Oota, Yoshikazu Kinoshita

**Affiliations:** 1 Department of Gastroenterology and Hepatology, Yokohama City University, 3-9 Fuku-ura, Kanazawa-ku, Yokohama, 236-0004 Japan; 2 Clinical Development Department, EA Pharma Co., Ltd., Tokyo, Japan; 3 Department of Gastroenterology and Hepatology, Shimane University School of Medicine, Izumo City, Shimane Japan

**Keywords:** Polyethylene glycol 3350 plus electrolytes, Chronic constipation, Prospective long-term clinical trial, Spontaneous bowel movement

## Abstract

**Background:**

Although polyethylene glycol 3350 plus electrolytes (PEG3350 + E) is the most widely used osmotic laxative in Europe, prospective data on its long-term (over 6 months) safety and efficacy are not available to date.

**Methods:**

Japanese patients with chronic constipation were randomized to receive PEG3350 + E or placebo for 2 weeks orally. Following this, the patients received PEG3350 + E in the 52-week extension study. The starting dose was 13.7 g/day dissolved in 125 mL of water, and dose titration was allowed (upper limit 41.1 g/day) according to the patient’s bowel condition. The primary efficacy endpoint was the change from baseline in frequency of spontaneous bowel movements (SBMs) at week 2 in the double-blind study. Secondary endpoints and adverse events were assessed. Safety and efficacy were also assessed in the extension study.

**Results:**

Among 204 patients who provided informed consent, 156 were randomized and included in the full analysis. The frequency of SBMs was significantly higher with PEG3350 + E [least squares mean (LSM) 4.3, 95% confidence interval (CI) 3.6–4.9] compared with placebo (LSM 1.6, 95% CI 1.2–2.1; *P* < 0.0001). A total of 153 patients entered the extension study; PEG3350 + E led to a sustained improvement in bowel function. The common adverse drug reactions during the entire study period were mild gastrointestinal disorders (abdominal pain 4.5%, diarrhea 3.8%, nausea 3.2%, abdominal distension 2.6%).

**Conclusions:**

Treatment with PEG3350 + E resolved constipation in the short term, was well tolerated, and led to sustained improvement in bowel function in the long-term treatment of Japanese patients with chronic constipation.

**Clinical trial registration number:**

Japic CTI-163167.

## Introduction

Chronic constipation (CC) is one of the most common chronic gastrointestinal symptoms. Patients with CC suffer from infrequent bowel movements, straining, sensation of incomplete evacuation, and hard stools. CC is most often defined by the Rome IV diagnostic criteria for functional constipation published in 2016 [1] (these criteria were unchanged from the 2006 version [2]). CC reportedly affects 14–17% of the population in Western countries [3, 4], occurring more frequently in women and the elderly [5]; it also affects both the physical and psychological quality of life [6]. About 28.4% of Japanese adults have self-reported constipation [7].

Current management for CC usually begins with lifestyle modifications, in which the physician increases the patient’s dietary fiber as well as fluid intake and amount of exercise. If these interventions are ineffective, stepwise drug therapy is used. The therapy usually begins with the administration of osmotic laxatives, including polyethylene glycol (PEG)-based laxatives that are currently used globally [8, 9].

In Europe, the first-line treatment for CC is polyethylene glycol 3350 plus electrolytes (PEG3350 + E), which is a minimally absorbable iso-osmotic agent with high molecular weight. It was reported that the mean urinary excretion of the administered PEG3350 dose ranged from 0.19 to 0.25% [10]. Clinical studies have demonstrated that electrolyte balance is maintained in patients treated with PEG3350 + E [11, 12]. The effect of PEG3350 + E on CC is due to its physicochemical property, which is unaffected by any ethnic factors. PEG3350 increases the water content of stool in a dose-dependent manner [13], which leads to improved colon motility of softened stools and defecation mechanics. PEG3350 + E has superior efficacy to lactulose, but with comparable tolerability to lactulose in adults and children [11, 12]. A number of independent systematic reviews have demonstrated the superior efficacy of PEG over lactulose [14, 15]. Although PEG3350 + E is the most widely used osmotic laxative in Europe, prospective clinical data on its long-term (over 6 months) safety and efficacy are not available to date [16].

In Japan, despite the introduction of novel pharmaceutics for CC (lubiprostone, linaclotide, and elobixibat) [17–20], PEG3350 + E has not been approved for use in clinical treatment, and magnesium oxide remains the most commonly used laxative for CC treatment. This phase 3 study aimed to compare the efficacy and safety of PEG3350 + E versus placebo for the treatment of Japanese patients with CC and to assess the long-term safety and efficacy of PEG3350 + E.

## Methods

### Study design

This phase 3 study comprised a confirmatory phase, followed by an extension phase. The confirmatory phase was a 2-week, multicenter, randomized, double-blind, placebo-controlled, parallel-group study, with an initial 2-week run-in period after screening and before treatment. The extension phase was a 52-week, open-label, single-arm study. The study was conducted between February 2016 and August 2017 (from the first informed consent to the last observation) at 17 medical institutions in Japan.

The study was performed in accordance with the ethical principles established in the Declaration of Helsinki and Good Clinical Practice guidelines. In addition, the study protocol and informed consent form were approved by the central institutional review boards of Yokohama Minoru Clinic, Shin-Nihonbashi Ishii Clinic, and Shinagawa East One Clinic. All patients provided written informed consent before study participation. For patients < 20 years old, the patients provided written informed assent and written informed consent was provided by the parents. Patient identification codes were used to enroll and identify patients. Adequate consideration was given to the protection of patients’ privacy.

### Patients

This study included male and non-pregnant female outpatients aged ≥ 15 years who satisfied the Rome III diagnostic criteria for functional constipation, which excludes recto-anal abnormalities. Patients with constipation-predominant irritable bowel syndrome (IBS-C) were also included in this study, in view of symptomatology and pathophysiology [21, 22]; latent class analysis suggests that functional constipation and IBS-C differ mostly in the severity, rather than the type, of symptoms [23]. All patients in the study met the inclusion criteria of spontaneous bowel movements (SBMs) occurring fewer than 3 times per week for at least 6 months, with fewer than 6 SBMs during the 2-week run-in period without loose stool (Bristol stool form scale [BSFS] type 6 and 7 [24]). In addition, one or more of the following symptoms had to be associated with at least 25% of SBMs for at least 6 months: straining, lumpy, or hard stools, and sensation of incomplete evacuation. Absence of organic lesions in the large intestine was also part of the inclusion criteria. Patients who had (or were suspected to have) organic constipation, drug-induced constipation, or constipation induced by disease, such as hypothyroidism or Parkinson’s disease, were excluded from the study.

After providing consent, patients underwent vital sign measurement and laboratory testing to confirm their eligibility, and were provisionally enrolled 15 days prior to the first day of treatment. Patient eligibility was further assessed based on the daily records of bowel movement during the 2-week screening period. Patients with organic constipation were excluded based on the results of colonoscopy performed at least 8 days prior to the screening period (for those patients who had not previously been ruled out because of colonoscopy or barium enema within the past 5 years).

Following the screening period, the electronic data capture system randomly assigned patients to either the PEG3350 + E or placebo group. The randomization process involved the use of a predetermined randomization table, which was created with a previously designed permuted block method. The randomization table was appropriately retained to ensure blinding of the study.

### Study treatment

PEG3350 + E (6.9 g sachet; Norgine Limited, Uxbridge, UK) is a powder formulation in sachets, each containing 6.5625 g PEG3350, 0.1754 g sodium chloride, 0.0893 g sodium bicarbonate, and 0.0251 g potassium chloride. The contents of 1 sachet were reconstituted in approximately 62.5 mL water. The investigational medicinal product was blinded by using a placebo powder that was indistinguishable from PEG3350 + E in terms of appearance, odor, volume, and appearance after dissolving in water.

In the confirmatory phase, patients were given 2 sachets of PEG3350 + E or placebo daily. The dose was increased by 2 sachets every other day until the stool was type 3, 4, or 5 on the BSFS. The maximum daily dose was 6 sachets. When patients’ BSFS scores were 6 or 7, drug administration was either temporarily suspended or continued with a 2-sachet dosage reduction. The dosage was increased again when the BSFS score became 1 or 2 or no SBM was observed during a single day.

The drugs were administered either once or twice a day depending on the dose as follows: 2 sachets/day, once at any time; 4 sachets/day, 2 sachets each in the morning and evening; 6 sachets/day, 2 sachets in the morning and 4 sachets in the evening. Rescue medication (bisacodyl suppository 10 mg) was allowed only for patients who experienced no bowel movement for at least 72 consecutive hours between the start of the run-in period and the last observation. In the extension phase, all patients received PEG3350 + E at the same dosage regimen of the confirmatory phase except in the following case: if the number of complete SBMs (CSBMs; defined as SBMs with a sense of complete evacuation) at 2 weeks just prior to each visit was ≥ 6, dosage was reduced by 2 sachets, or the treatment was suspended at the physician’s discretion. After suspension, if the weekly number of SBMs was < 3, treatment was resumed at the same dosage as before suspension.

### Study assessments

After randomization, patients received the appropriate treatment from days 1 to 14 and visited the hospital at weeks 1 and 2. To evaluate efficacy, patients’ electronic diaries were used to investigate the date and time of bowel movements, stool consistency, sensation of incomplete evacuation, date and time of rescue medication, and number of study treatment sachets used in the morning and evening. Stool consistency was self-assessed by patients on a scale from type 1 (hard lumps) to type 7 (liquid consistency) according to the BSFS. Prior to the beginning of the extension phase, treatment was suspended for 2 weeks in order to secure the data obtained during the confirmatory phase. In the extension phase, patients visited the hospital at weeks 0, 2, and 4, and every 4 weeks thereafter.

Vital signs (pulse, blood pressure, and weight) were also assessed at baseline and week 2 in the confirmatory phase; at weeks 0, 2, and 4; and every 4 weeks thereafter in the extension phase. Standard laboratory tests for hematology, biochemistry, and urinalysis were performed at baseline and week 2 in the confirmatory phase, and at weeks 0, 4, 12, 24, 36, and 52 or at the time of discontinuation in the extension phase.

### Study endpoints

The primary endpoint of the confirmatory phase was the change in the frequency of SBMs from baseline at week 2 (i.e., the last week of the 2-week run-in period) of treatment. The secondary endpoints were the change in the frequency of SBMs and CSBMs from baseline at week 1, change in the frequency of CSBMs from baseline at week 2, proportion of SBM and CSBM responders (defined as ≥ 3 SBMs/CSBMs and increase of at least 1 SBM/CSBM per week from baseline), and median number of days to first SBM and CSBM. Other secondary endpoints were the use of rescue medication, stool consistency (using BSFS), and the number of sachets of study drugs used. The efficacy endpoints of the extension phase were relative to baseline, as with the confirmatory phase. In addition, the number of days during which the treatment was suspended was recorded.

Safety endpoints included adverse events (AEs) coded according to the Medical Dictionary for Regulatory Activities (MedDRA) version 18.1, results from laboratory tests, and vital signs observed during the time including the confirmatory and extension phases and the period between the two.

Post hoc analysis of the extension phase included the following assessments: the median weekly frequency of each BSFS type, duration of SBMs measured every 3 h, and length of treatment cessation in patients whose medical condition improved temporarily.

### Sample size design and statistical analyses

We planned to include 70 patients in each group for this study. This was based on the differences in the primary endpoint (change in SBM frequency from baseline at week 2) between the 2 groups (3.10 times/week) and their standard deviations (SDs; placebo, 5.15 SBMs/week; PEG3350 + E, 6.07 SBMs/week) in the prior phase 2 study (91020/2 study, Norgine B.V.). A sample size of 61 patients per treatment group was estimated to provide more than 90% power to test the hypothesis that there is a difference in the primary endpoint between the 2 groups, with a 2-sided *α* of 0.05, based on a *t* test with unequal variances. Assuming withdrawals, we planned to include an additional 10% of patients in each treatment group.

All efficacy analyses were based on the modified intent-to-treat population without imputation for any missing data. Multiplicity of endpoints was not accounted for in the analyses. Analysis of changes from baseline in SBM/CSBM was performed using analysis of covariance with baseline value as covariate, assuming unequal variances. At the week of discontinuation, if a patient had < 5 days of diary entries regarding defecation during a week, that week was considered not assessable and was treated as a missing value. Fisher’s exact test was used for the comparison of the proportion of patients who were SBM/CSBM responders between the treatment groups. Wilson’s score method was used to calculate the 95% confidence intervals (CIs). The median time to first SBM/CSBM was assessed using the Kaplan–Meier method, and the log-rank test was used for pairwise comparisons. Wilcoxon rank-sum test was used to determine the differences between the groups (in the confirmatory phase) and time points (in the extension phase).

The safety analysis population included all patients who received at least one dose of the study drug. The numbers and proportions of patients who had adverse drug reactions (ADRs) were summarized by treatment group.

All reported *P* values were based on 2-sided tests, and the significance level was set at 0.05. All data were analyzed using SAS 9.3 (SAS Institute Inc., Cary, NC, USA) by A2 Healthcare Co., Ltd. (Tokyo, Japan).

## Results

### Patient baseline characteristics

**Fig. 1:**
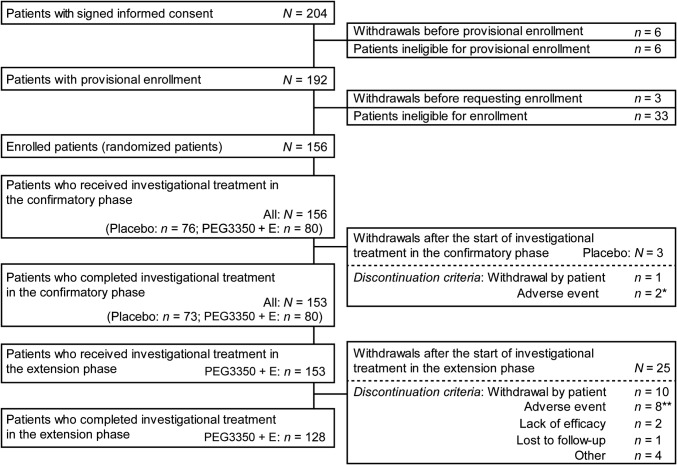
Patient disposition. *PEG3350 *+ *E* polyethylene glycol 3350 plus electrolytes. *n = 2; one subject each experienced vertigo positional and contusion. **n = 8; one subject each experienced infectious colitis, breast cancer, insomnia, abdominal discomfort, constipation, nausea, eczema, and erythema

A total of 156 patients were administered either PEG3350 + E (*n* = 80) or placebo (*n* = 76) in the confirmatory phase (Fig. 1). All 156 patients received at least one dose of the drug and were included in the modified intent-to-treat and safety populations. Baseline demographic characteristics in both groups were similar and well balanced (Table 1).

**Table 1:** Patient baseline characteristics

	Confirmatory phase		All (*n* = 156)
	Placebo (*n* = 76)	PEG3350 + E (*n* = 80)	
Age (years)	42.0 ± 12.8	44.3 ± 11.6	43.2 ± 12.2
Age ≥ 65 years	5 (6.6%)	4 (5.0%)	9 (5.8%)
Female sex	61 (80.3%)	71 (88.8%)	132 (84.6%)
Body mass index (kg/m^2^)	21.77 ± 2.97	21.64 ± 2.94	21.70 ± 2.95
Fulfilled criteria for IBS-C	10 (13.2%)	15 (18.8%)	25 (16.0%)
SBMs per week^a^	1.4 ± 0.9	1.6 ± 0.9	1.5 ± 0.9
CSBMs per week^a^	0.4 ± 0.7	0.4 ± 0.7	0.4 ± 0.7
Use of rescue medication^a^	18 (23.7%)	17 (21.3%)	35 (22.4%)
Stool consistency score^b^	2.2 ± 1.1	2.1 ± 1.0	2.1 ± 1.1

Data are mean ± SD or *n* (%)

*CSBM c*omplete spontaneous bowel movement, *IBS*-*C* constipation-predominant irritable bowel syndrome, *PEG3350 *+ *E* polyethylene glycol 3350 plus electrolytes, *SBM* spontaneous bowel movement, *SD* standard deviation

^a^Baseline value was based on week 2 of the run-in period

^b^Stool consistency was assessed using the Bristol stool form scale

### Confirmatory phase

A significantly higher increase in the primary endpoint of change in the frequency of SBMs from baseline at week 2 was observed in the PEG3350 + E group [LSM (SE) 4.3 (0.2), 95% CI 3.6–4.9] than in the placebo group [LSM (SE) 1.6 (0.3), 95% CI 1.2–2.1; *P* < 0.0001; Table 2]. Other SBM-related endpoints, including change in the frequency of SBMs from baseline at week 1 and the proportion of responders at weeks 1 and 2, were also higher in the PEG3350 + E group (Table 2). Median number of days to first SBM was similar in both groups.

**Table 2:** Efficacy in the confirmatory phase

	Placebo (*n* = 76)	PEG3350 + E (*n* = 80)	Difference between groups	*P* value
**Primary endpoint**				
Change in SBMs during week 2 compared with baseline, LSM (SE) [95% CI]	1.62 (0.24) [1.15–2.09]	4.27 (0.32) [3.63–4.92]	2.66 (0.40) [1.86–3.54]	< 0.0001^a^
**Secondary endpoints**				
SBMs				
Change in SBMs during week 1 compared with baseline, mean (SE) [95% CI]	1.35 (0.22) [0.92–1.79]	3.36 (0.28) [2.81–3.92]	2.01 (0.35) [1.31–2.71]	< 0.0001^a^
Weekly responders at week 1, *n* (%)	38 (50.0%)	64 (80.0%)		< 0.0001^b^
Weekly responders at week 2, *n* (%)	41 (56.2%)	69 (86.3%)		< 0.0001^b^
Time to first SBM using the Kaplan–Meier method, days, median (95% CI)	2.0 (2.0–3.0)	2.0 (2.0–3.0)		0.0757^c^
CSBMs				
Change in CSBMs during week 1 compared with baseline, mean (SE) [95% CI]	0.74 (0.16) [0.42–1.06]	1.22 (0.19) [0.85–1.60]	0.49 (0.25) [0.00–0.98]	0.0516^a^
Change in CSBMs during week 2 compared with baseline, mean (SE) [95% CI]	0.92 (0.19) [0.54–1.30]	1.76 (0.25) [1.27–2.25]	0.84 (0.31) [0.22–1.46]	0.0082^a^
Weekly responders at week 1, *n* (%)	15 (19.7%)	19 (23.8%)		0.5667^b^
Weekly responders at week 2, *n* (%)	18 (24.7%)	30 (37.5%)		0.1162^b^
Time to first CSBM using the Kaplan–Meier method, days, median (95% CI)	9 (5.0–)	6 (4.0–7.0)		0.0293^c^
Bristol stool form scale				
Stool consistency score at week 1, mean ± SD	2.94 ± 1.15	3.85 ± 1.00		
	[1, 2] 29 (42.0%) [3, 4, 5] 40 (58.0%) [6, 7] 0 (0%)	[1, 2] 7 (8.9%) [3, 4, 5] 69 (87.3%) [6, 7] 3 (3.8%)		< 0.0001^d^
Stool consistency score at week 2, mean ± SD	3.34 ± 1.28	4.34 ± 0.96		
	[1, 2] 19 (29.2%) [3, 4, 5] 43 (66.2%) [6, 7] 3 (4.6%)	[1, 2] 7 (8.8%) [3, 4, 5] 64 (80.0%) [6, 7] 9 (11.3%)		< 0.0001^d^

CSBMs were defined as SBMs with a sense of complete evacuation. Responders were defined as patients with 3 or more BMs and an increase of at least 1 BM per week from baseline

*ANCOVA* analysis of covariance, *BM* bowel movement, *CI* confidence interval, *CSBM* complete spontaneous bowel movement, *LSM* least squares mean, *PEG3350 *+ *E* polyethylene glycol 3350 plus electrolytes*, SBM* spontaneous bowel movement, *SD* standard deviation, *SE* standard error

^a^ANCOVA

^b^Fisher’s exact test

^c^Log-rank test

^d^Wilcoxon rank sum test

For CSBM-related endpoints, the change in the frequency of CSBMs from baseline at week 2 was higher with PEG3350 + E than with placebo. Median number of days to first CSBM was shorter in the PEG3350 + E group (Table 2). However, the change in the frequency of CSBMs from baseline at week 1 and the proportion of responders did not differ between the groups. Stool consistency was greater with PEG3350 + E (Table 2).

At baseline, 17 (21%) patients in the PEG3350 + E group and 18 (24%) in the placebo group required rescue medication. At week 2, in the confirmatory phase, 4 (5%) patients in the PEG3350 + E group and 11 (15%) in the placebo group required such treatment.

The mean ± SD dose of drug administered at week 1 and week 2 of the confirmatory phase was 20.73 ± 6.77 and 24.18 ± 11.58 sachets in the PEG3350 + E group and 24.67 ± 6.19 and 34.67 ± 10.83 sachets in the placebo group, respectively.

### Extension phase

**Fig. 2:**
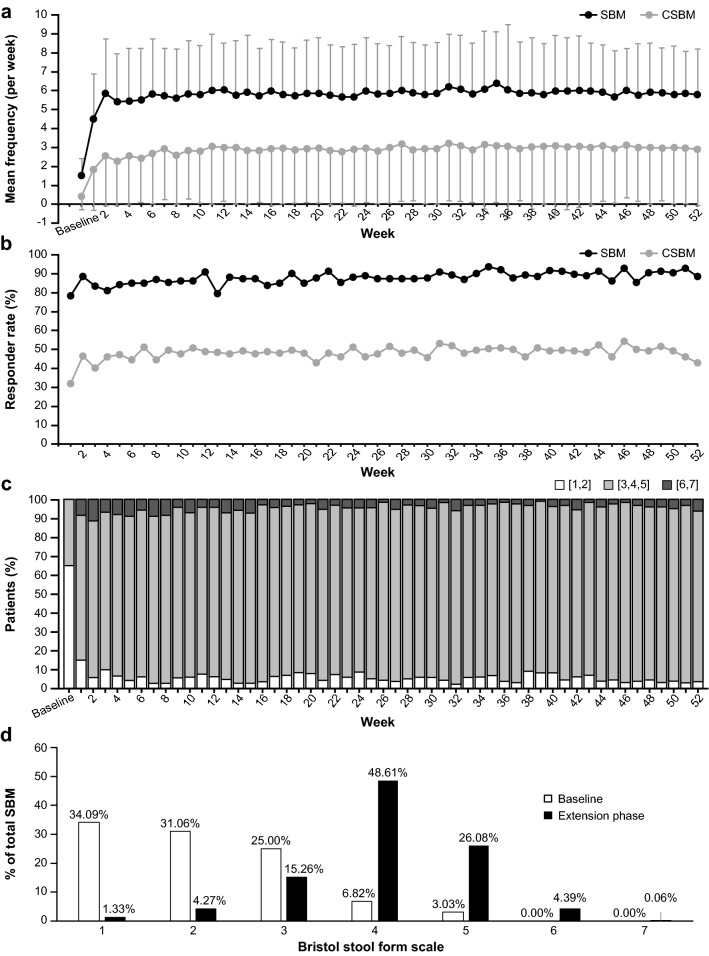
Effects of PEG3350 + E in the extension phase. **a** Mean frequency of SBMs and CSBMs. **b** SBM and CSBM responder rate. **c** Ratio of type of stool consistency (median) categorized using the Bristol stool form scale [1, 2], [3, 4, 5], and [6, 7]. **d** Ratio of stool consistency (weekly median) at baseline and in the extension phase. *BM* bowel movement, *CSBM* complete spontaneous bowel movement, *PEG3350 *+ *E* polyethylene glycol 3350 plus electrolytes, *SBM* spontaneous bowel movement. Baseline mean was based on week 2 in the run-in period. CSBMs were defined as SBMs with a sense of complete evacuation. Responders were defined as patients with 3 or more BMs and an increase of at least 1 BM per week from baseline

The mean number of weekly SBMs and CSBMs numerically and significantly increased from baseline throughout the 52-week treatment period (Fig. 2a). The proportion of weekly SBM or CSBM responders did not change throughout the 52-week study period (Fig. 2b). The median weekly stool consistency was 2.14 ± 1.06 (mean ± SD, hereafter the same) at baseline and 3.93 ± 1.25 at week 2 of the extension phase. This remained stable in the range of 3.91–4.36 during week 1 through week 52 (data not shown). When the stool consistency based on BSFS was classified into score groups (1, 2), (3, 4, 5), or (6, 7), the ratio of (1, 2) decreased from week 1 of the extension phase, while the ratio of (3, 4, 5) and (6, 7) increased. The category (6, 7) remained stable from week 3 of the extension phase (Fig. 2c). It was significantly different at weeks 24 and 52 of the extension phase versus baseline (Wilcoxon signed-rank test, *P* < 0.0001). The post hoc analysis showed that the ratio of BSFS type 4, type 1, and type 7 in the extension phase was 48.61, 1.33, and 0.06%, respectively (Fig. 2d).

**Fig. 3:**
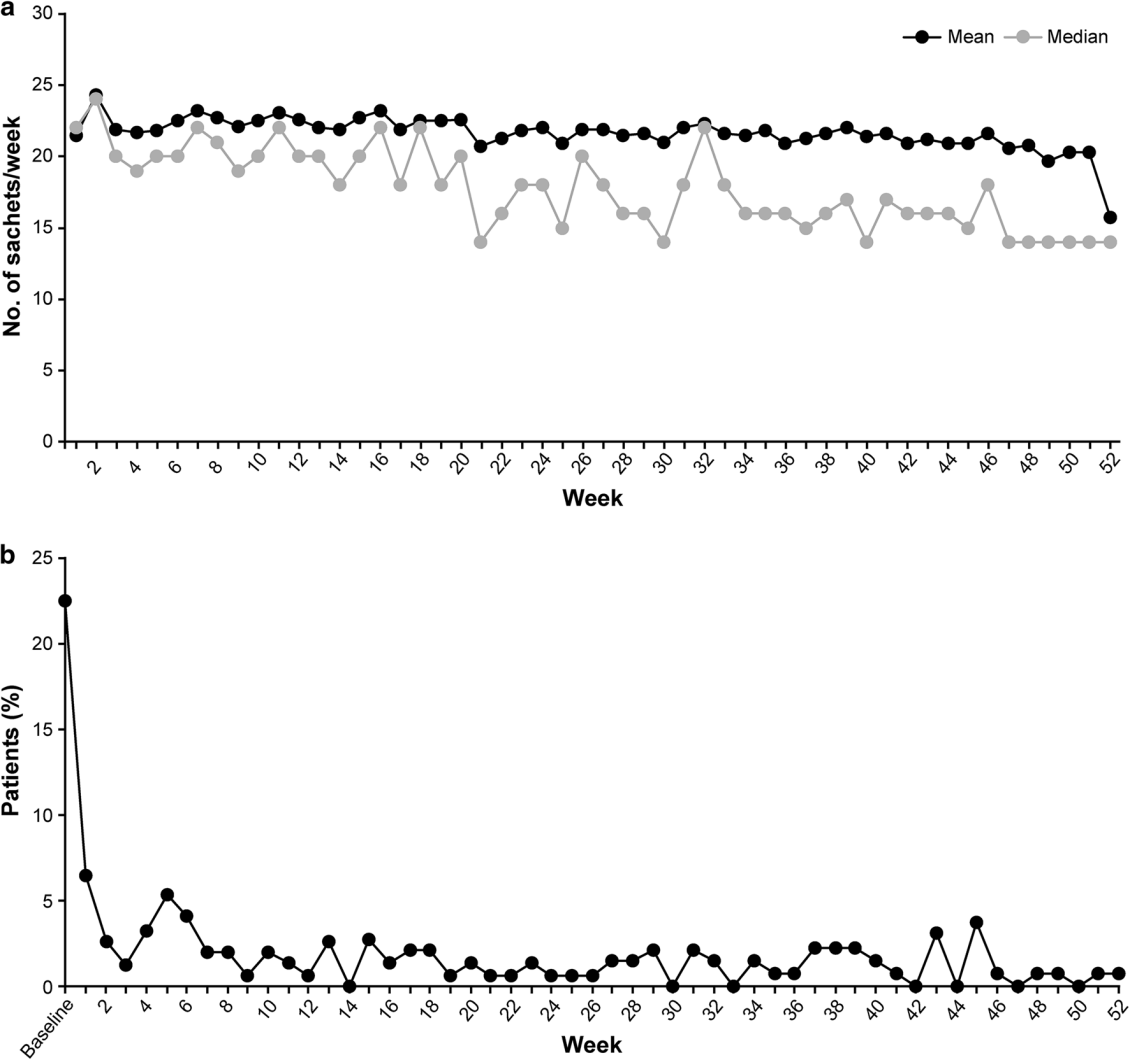
PEG3350 + E and rescue medication use in the extension phase. **a** Mean and median number of weekly sachets. **b** Ratio of rescue medication use. *PEG3350 *+ *E* polyethylene glycol 3350 plus electrolytes. Baseline mean was based on week 2 in the run-in period

While the mean number of sachets of drug prescribed remained at around 19.7–24.3 sachets (2.8–3.5 sachets/day; Fig. 3a) during the period between week 1 and week 51, it decreased to 15.7 sachets (2.2 sachets/day) at week 52. The number of patients who required rescue medication was 35 (22.4%) at baseline (Fig. 3b), and decreased markedly as the study progressed. Rescue medication was taken by 10 (7%) patients in the first week, and approximately 0 (0%) to 8 (5%) patients throughout 52 weeks.

**Fig. 4:**
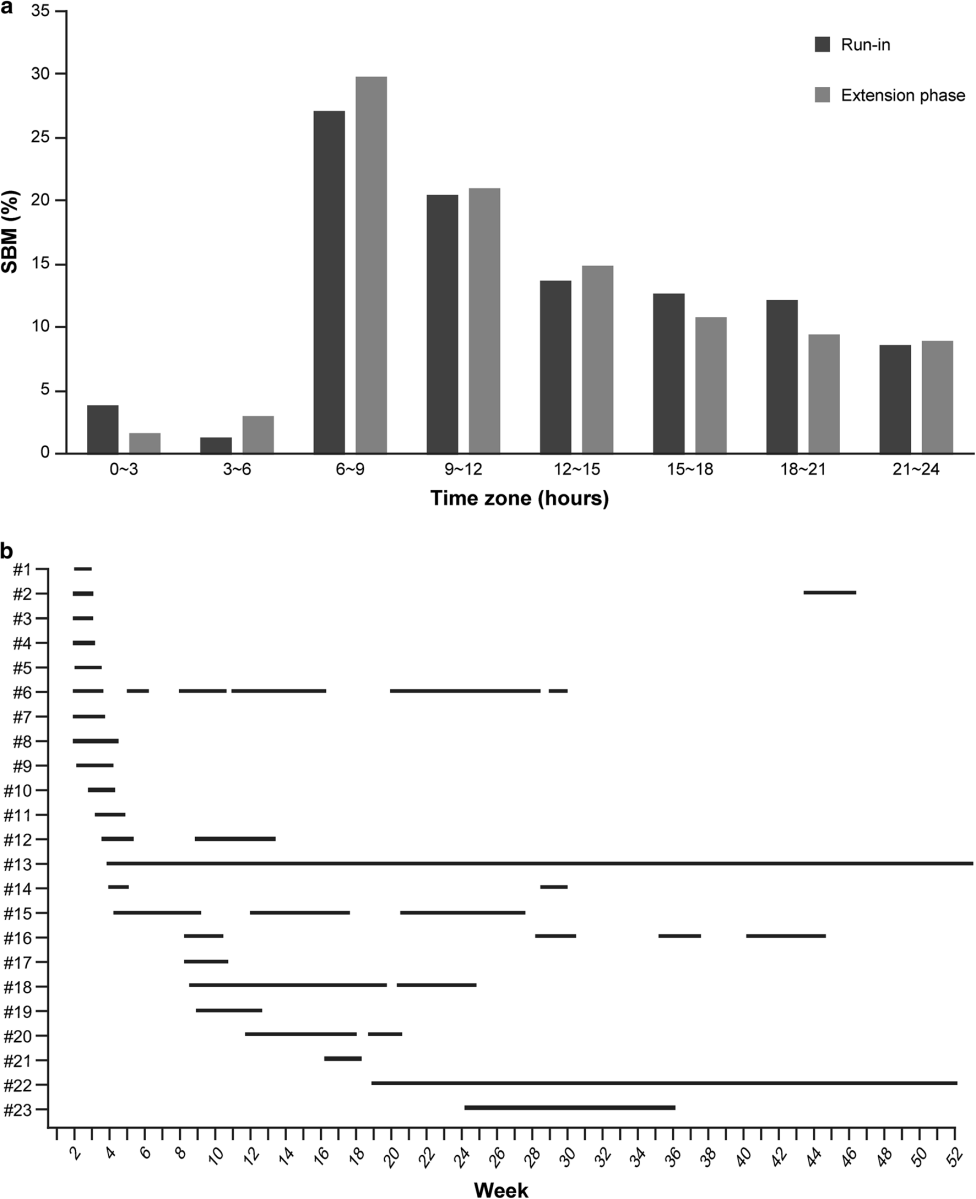
Other post hoc analyses. **a** Time zone ratio of SBM in the run-in period compared with the extension phase. **b** Individual data for treatment suspension as a result of improvement. *Y*-axis shows data for each patient. *SBM* spontaneous bowel movement

In the post hoc analysis, the time zone ratio of SBM every 3 h was not influenced by PEG3350 + E, and the prime-time zone of SBM was 6 a.m. to 9 a.m. (Fig. 4a). A total of 143 patients discontinued treatment for 55.2 ± 80.4, 17.0 days (mean ± SD, median, hereafter the same) during the extension phase. Twenty-three patients stopped medication as a result of improvement for 60.2 ± 84.1, 17.0 days. Subsequently, 21 of these patients were re-administered PEG3350 + E (Fig. 4b).

### Safety

The safety analysis set from the confirmatory phase included 156 patients (PEG3350 + E group, 80; placebo group, 76) and that of the extension phase included 153 patients. The summary of AEs during the extension phase was conducted for the entire study period (from the confirmatory phase to week 52 of the extension phase). The summary of ADRs is shown in Table 3. In the confirmatory phase, the incidence rate of AEs was 20.0% (16 of 80 patients) in the PEG3350 + E group and 19.7% (15 of 76 patients) in the placebo group. The incidence rate of ADRs was 7.5% (6 of 80 patients) and 5.3% (4 of 76 patients) in the PEG3350 + E and placebo groups, respectively. The incidence rate of AEs and ADRs in the entire study period was 78.8% (123 of 156 patients) and 21.2% (33 of 156 patients), respectively. All ADRs were mild in severity.

**Table 3:** Summary of ADRs

	Confirmatory phase		Entire period
	Placebo	PEG3350 + E	All
	*n* = 76	*n* = 80	*N* = 156
AEs, *n* (%)	15 (19.7)	16 (20.0)	123 (78.8)
ADRs, *n* (%)	4 (5.3)	6 (7.5)	33 (21.2)
Mild	4 (5.3)	6 (7.5)	33 (21.2)
ADRs leading to discontinuation, *n* (%)	0 (0.0)	1 (1.3)	4 (2.6)
ADRs for ≥ 2% of patients, *n* (%)			
Abdominal pain	1 (1.3)	1 (1.3)	7 (4.5)
Diarrhea	0 (0.0)	1 (1.3)	6 (3.8)
Nausea	1 (1.3)	1 (1.3)	5 (3.2)
Abdominal distension	0 (0.0)	2 (2.5)	4 (2.6)

Categorization of AEs was based on the Medical Dictionary for Regulatory Activities version 18.1

*ADR* adverse drug reaction, *AE* adverse event, *PEG3350 *+ *E* polyethylene glycol 3350 plus electrolytes

The most common ADR in the confirmatory phase was abdominal distension reported in 2.5% (2 of 80) and 0.0% (0 of 76) of patients in the PEG3350 + E and placebo groups, respectively. The most common ADRs during the entire study period were mild gastrointestinal disorders (abdominal pain 4.5%; diarrhea 3.8%; nausea 3.2%; and abdominal distension 2.6%).

The summary of AEs of the entire period included AEs reported in patients in the placebo group during the confirmatory phase and in all patients during the 2-week break between the confirmatory and extension phases. Excluding these AEs, a common AE in the PEG3350 + E group was nasopharyngitis, and common ADRs were mild gastrointestinal disorders such as abdominal pain and diarrhea.

The entire study period from the confirmatory phase day 1 was classified into periods of 90 days. An AE that was first observed at a rate of 5% or higher in any period was nasopharyngitis; its incidence in the period days 1–90, days 91–180, days 180–270, days 271–360, and day 361 onward was 14.7% (23 of 156 patients), 8.3% (13 of 156 patients), 9.0% (14 of 156 patients), 1.3% (2 of 156 patients), and 1.3% (2 of 156 patients), respectively. No ADR occurred at a rate of 5% or higher in either the period or treatment group.

During the confirmatory phase, no fatal or non-fatal serious AEs were reported in either group. Throughout the entire study period, there were no deaths. The incidence of non-fatal serious AEs observed following treatment with PEG3350 + E was 1.9% (3 of 156 patients; 4 events). These were infectious colitis, breast cancer, retinal detachment, and macular hole, each of which was observed in 1 patient. A causal relationship with the study drug was ruled out for all these cases. The incidence rate of ADRs leading to drug discontinuation during the confirmatory phase was 1.3% (1 of 80 patients; eczema) in the PEG3350 + E group. Throughout the entire study period, 2.6% (4 of 156; abdominal discomfort, nausea, eczema, and erythema) of patients experienced ADRs that led to drug discontinuation. There were no clinically significant changes in clinical laboratory values and vital signs during either the confirmatory or extension phase.

## Discussion

The results from the confirmatory phase showed that PEG3350 + E was more effective and well tolerated for the short-term treatment of Japanese patients with CC compared with placebo, using the same dosage that has been used in Europe (13.7–41.1 g/day). This double-blind study was of short duration, but it was reported that the mean number of successful defecations increased from 2 weeks following the drug administration and were sustained through the 12-week study period [25]; similar results were obtained in the extension phase.

During the confirmatory phase, the dosage of PEG3350 + E increased from week 1 (20.73 ± 6.77 [mean ± SD]) to week 2 (24.18 ± 11.58). At week 1, a larger increase in SBM frequency from the baseline was observed in patients in the PEG3350 + E group than those in the placebo group. Furthermore, PEG3350 + E showed a greater increase in stool consistency at week 1 compared with placebo. Treatment with PEG3350 + E maintained patients’ stool consistency equivalent to that of type 4 stool defined in BSFS. These observations suggest that PEG3350 + E increased SBMs and improved stool consistency from the first treatment week.

In this study, the findings from the extension phase showed that PEG3350 + E is well tolerated. A frequently observed AE in the PEG3350 + E group was nasopharyngitis; however, cases of nasopharyngitis did not increase throughout the study period. Common ADRs observed were mild gastrointestinal disorders, including abdominal pain and diarrhea. These ADRs are thought to be caused by the mechanism of action of PEG3350 + E.

PEG3350 + E improved bowel function throughout the extension phase. Under the dosage regimen that has been commonly used in Europe and without any increase in dosage, administering PEG3350 + E increased not only the frequency of bowel movements but also the stool consistency of Japanese patients with CC.

The dosage of PEG3350 + E was adjusted so that patients’ stool consistency remained at a level defined as BSFS type 3, 4, or 5. Thus, the median weekly BSFS was maintained at about type 4 throughout the experimental period. However, importantly, good stool consistency (BSFS type 3, 4, or 5) was recorded during more than 80% of the treatment period, while hard stool (BSFS type 1 or 2) and loose stool (BSFS type 6 or 7) were recorded during less than 10% of the treatment period, except for treatment weeks 1 and 2. To clarify the efficacy of PEG3350 + E for stool consistency, a post hoc analysis was conducted. Accumulated and classified stool consistency records showed that BSFS type 4 was the most observed and type 1 (separate, hard, lumpy) and type 7 (watery) were the least observed (Fig. 2d). Moreover, dosage of PEG3350 + E was almost stable or slightly reduced in long-term use.

The mean number of sachets of the study drug used at week 52 was lesser than in other weeks (Fig. 3a) because some patients visited a hospital before the planned visiting day (day 365) using visit allowance (± 7 days) and their treatment duration was less than 7 days. This study reconfirmed that dosage adjustment of PEG3350 + E using BSFS is very useful in controlling the stool consistency as well as the increment of SBM.

Our study indicated the necessity of a long-term treatment for the complete treatment of CC. Only 2 out of 153 patients remained completely treatment-free after a short treatment period, suggesting the need for long-term treatment and a laxative with high tolerability and prolonged effects, such as PEG3350 + E, to achieve complete treatment effects in CC.

The data from patients’ electronic diaries, namely, time zone ratio of SBM every 3 h, were not influenced by PEG3350 + E. These results suggest that PEG3350 + E induces natural bowel movement without trigger action for bowel movement.

The data on long-term use of PEG3350 + E for CC are limited. PEG3350 + E was administered for 6 months in an earlier prospective study; however, in that study, patients were hospitalized in a single medical facility and the PEG3350 + E formulation used contained sodium sulfate [16]. The longest duration of treatment with PEG3350 + E of the same formulation as used in this study is 3 months [26]. According to a previously conducted retrospective study, the longest duration of PEG3350 + E administration is 24 months; however, the study was limited to data collected from hospitalized patients with severe learning disabilities [27]. In contrast, this clinical trial was prospective and conducted with outpatients recruited from various sites. Thus, despite the limitations of this being an open-label, single-arm study, our results sufficiently demonstrate the safety and effects of long-term use of PEG3350 + E in Japanese patients with CC.

In conclusion, the results from the 2-week confirmatory phase demonstrated that PEG3350 + E is significantly more effective than placebo in treating CC. Moreover, the efficacy of the drug lasted for the entire 52-week extension phase, and PEG3350 + E was shown to have high tolerability in the long-term treatment of CC.

## References

[1] Mearin F, Lacy BE, Chang L (2016). Bowel disorders. Gastroenterology.

[2] Longstreth GF, Thompson WG, Chey WD (2006). Functional bowel disorders. Gastroenterology.

[3] Suares NC, Ford AC (2011). Prevalence of, and risk factors for, chronic idiopathic constipation in the community: systematic review and meta-analysis. Am J Gastroenterol.

[4] Peppas G, Alexiou VG, Mourtzoukou E (2008). Epidemiology of constipation in Europe and Oceania: a systematic review. BMC Gastroenterol.

[5] Higgins PD, Johanson JF (2004). Epidemiology of constipation in North America: a systematic review. Am J Gastroenterol.

[6] Wald A, Scarpignato C, Kamm MA (2007). The burden of constipation on quality of life: results of a multinational survey. Aliment Pharmacol Ther.

[7] Tamura A, Tomita T, Oshima T (2016). Prevalence and self-recognition of chronic constipation: results of an internet survey. J Neurogastroenterol Motil.

[8] Lindberg G, Hamid SS, Malfertheiner P (2011). World Gastroenterology Organisation global guideline: constipation—a global perspective. J Clin Gastroenterol.

[9] Bharucha AE, Dorn SD, Lembo A, American Gastroenterological Association (2013). American Gastroenterological Association medical position statement on constipation. Gastroenterology.

[10] Pelham RW, Nix LC, Chavira RE (2008). Clinical trial: single- and multiple-dose pharmacokinetics of polyethylene glycol (PEG-3350) in healthy young and elderly subjects. Aliment Pharmacol Ther.

[11] Attar A, Lémann M, Ferguson A (1999). Comparison of a low dose polyethylene glycol electrolyte solution with lactulose for treatment of chronic constipation. Gut.

[12] Candy DC, Edwards D, Geraint M (2006). Treatment of faecal impaction with polyethylene glycol plus electrolytes (PGE + E) followed by a double-blind comparison of PEG + E versus lactulose as maintenance therapy. J Pediatr Gastroenterol Nutr.

[13] Hammer HF, Santa Ana CA, Schiller LR (1989). Studies of osmotic diarrhea induced in normal subjects by ingestion of polyethylene glycol and lactulose. J Clin Investig.

[14] Lee-Robichaud H, Thomas K, Morgan J (2010). Lactulose versus polyethylene glycol for chronic constipation. Cochrane Database Syst Rev.

[15] Candy D, Belsey J (2009). Macrogol (polyethylene glycol) laxatives in children with functional constipation and faecal impaction: a systematic review. Arch Dis Child.

[16] Paille F, Colombey N, Alleaume B (1999). An open six-month study of the safety of Transipeg for treating constipation in community medicine. J Drug Assess.

[17] Fukudo S, Hongo M, Kaneko H (2015). Lubiprostone increases spontaneous bowel movement frequency and quality of life in patients with chronic idiopathic constipation. Clin Gastroenterol Hepatol.

[18] Fukudo S, Miwa H, Nakajima A (2019). High-dose linaclotide is effective and safe in patients with chronic constipation: a phase III randomized, double-blind, placebo-controlled study with a long-term open-label extension study in Japan. Neurogastroenterol Motil.

[19] Nakajima A, Seki M, Taniguchi S (2018). Determining an optimal clinical dose of elobixibat, a novel inhibitor of the ileal bile acid transporter, in Japanese patients with chronic constipation: a phase II, multicenter, double-blind, placebo-controlled randomized clinical trial. J Gastroenterol.

[20] Nakajima A, Seki M, Taniguchi S (2018). Safety and efficacy of elobixibat for chronic constipation: results from a randomised, double-blind, placebo-controlled, phase 3 trial and an open-label, single-arm, phase 3 trial. Lancet Gastroenterol Hepatol.

[21] Shekhar C, Monaghan PJ, Morris J (2013). Rome III functional constipation and irritable bowel syndrome with constipation are similar disorders within a spectrum of sensitization, regulated by serotonin. Gastroenterology.

[22] Zhao YF, Ma XQ, Wang R (2011). Epidemiology of functional constipation and comparison with constipation-predominant irritable bowel syndrome: the Systematic Investigation of Gastrointestinal Diseases in China (SILC). Aliment Pharmacol Ther.

[23] Koloski NA, Jones M, Young M (2015). Differentiation of functional constipation and constipation predominant irritable bowel syndrome based on Rome III criteria: a population-based study. Aliment Pharmacol Ther.

[24] Heaton KW, Radvan J, Cripps H (1992). Defecation frequency and timing, and stool form in the general population: a prospective study. Gut.

[25] Hardikar W, Cranswick N, Heine RG (2007). Macrogol 3350 plus electrolytes for chronic constipation in children, a single-centre, open-label study. J Paediatr Child Health.

[26] Ferguson A, Culbert P, Gillett H (1999). New polyethylene glycol electrolyte solution for the treatment of constipation and faecal impaction. Ital J Gastroenterol Hepatol..

[27] Migeon-Duballet I, Chabin M, Gautier A (2006). Long-term efficacy and cost-effectiveness of polyethylene glycol 3350 plus electrolytes in chronic constipation: a retrospective study in a disabled population. Curr Med Res Opin.

